# Antimicrobial susceptibility testing of rapidly growing mycobacteria isolated in Japan

**DOI:** 10.1186/s12879-017-2298-8

**Published:** 2017-03-07

**Authors:** Shuji Hatakeyama, Yuki Ohama, Mitsuhiro Okazaki, Yoko Nukui, Kyoji Moriya

**Affiliations:** 1 Division of General Internal Medicine, Jichi Medical University Hospital, Yakushiji, Shimotsuke-shi, Tochigi 329-0498 Japan; 2 Division of Infectious Diseases, Jichi Medical University Hospital, Yakushiji, Shimotsuke-shi, Tochigi 329-0498 Japan; 3 Department of Infectious Diseases, The University of Tokyo Hospital, Hongo, Bunkyo-ku, Tokyo, 113-8655 Japan; 4 Department of Infection Control and Prevention, The University of Tokyo Hospital, Hongo, Bunkyo-ku, Tokyo, 113-8655 Japan; 5 Department of Medical Technology, School of Health Sciences, Tokyo University of Technology, Nishikamata, Ota-ku, Tokyo, 144-8535 Japan; 6 Department of Infection Control and Prevention, Tokyo Medical and Dental University, Yushima, Bunkyo-ku, Tokyo, 113-8510 Japan

**Keywords:** Rapidly growing mycobacteria, *Mycobacterium abscessus* complex, Antimicrobial susceptibility, Tigecycline, Tebipenem, Macrolides

## Abstract

**Background:**

Difficult-to-treat infections caused by rapidly growing mycobacteria (RGM) are increasingly observed in clinical settings. However, studies on antimicrobial susceptibilities and effective treatments against RGM in Japan are limited.

**Methods:**

We conducted susceptibility testing of potential antimicrobial agents, including tigecycline and tebipenem, against RGM. Clinical RGM isolates were collected from a university hospital in Japan between December 2010 and August 2013. They were identified using matrix-assisted laser desorption/ionization time-of-flight mass spectrometry and the sequencing of 16S rRNA, *rpoB*, and *hsp65* genes. The samples were utilized for susceptibility testing using 16 antimicrobials, with frozen broth microdilution panels.

**Results:**

Forty-two isolates were obtained: 13, *Mycobacterium abscessus* complex; 12, *Mycobacterium chelonae*; 9, *Mycobacterium fortuitum*; and 8, *M. fortuitum* group species other than *M. fortuitum*. Different antimicrobial susceptibility patterns were observed between RGM species. Clarithromycin-susceptible strain rates were determined to be 0, 62, and 100% for *M. fortuitum*, *M. abscessus* complex, and *M. chelonae*, respectively. *M. abscessus* complex (100%) and >80% *M. chelonae* isolates were non-susceptible, while 100% *M. fortuitum* group isolates were susceptible to moxifloxacin. Linezolid showed good activity against 77% *M. abscessus* complex, 89% *M. fortuitum*, and 100% *M. chelonae* isolates. Regardless of species, all tested isolates were inhibited by tigecycline at very low minimal inhibitory concentrations (MICs) of ≤0.5 μg/mL. MICs of tebipenem, an oral carbapenem, were ≤4 μg/mL against all *M. fortuitum* group isolates.

**Conclusions:**

Our study demonstrates the importance of correct identification and antimicrobial susceptibility testing, including the testing of potential new agents, in the management of RGM infections.

## Background

Rapidly growing mycobacteria (RGM) can cause various diseases in humans, including lung, skin and soft tissue, bone, and catheter-related blood stream infections, as well as disseminated infections. *Mycobacterium abscessus* complex, *Mycobacterium chelonae*, and *Mycobacterium fortuitum* complex are the most commonly encountered RGM strains clinically. Different strains belonging to the *M. fortuitum* group and *Mycobacterium smegmatis* and *Mycobacterium mucogenicum* group strains, which can cause human infections, account for <10% of rapidly growing mycobacterial infections [1, 2].

Recently, *M. abscessus* complex was divided into three subspecies, *M. abscessus* subsp. *abscessus*, *M. abscessus* subsp. *bolletii*, and *M. abscessus* subsp. *massiliense*, based on the multiple sequencing analyses of 16S rRNA and several housekeeping genes, such as *rpoB*, *hsp65*, *secA*, and *sodA. M. abscessus* subsp. *abscessus* and *M. abscessus* subsp. *bolletii* usually possess an inducible macrolide resistant gene, unlike *M. abscessus* subsp. *massiliense* [3]. *M. fortuitum* group comprises of more than 10 species, including *M. fortuitum*, *Mycobacterium peregrinum*, *Mycobacterium senegalense*, *Mycobacterium septicum*, *Mycobacterium alvei*, *Mycobacterium houstonense*, *Mycobacterium boenickei*, *Mycobacterium conceptionense*, *Mycobacterium porcinum*, *Mycobacterium neworleansense*, *Mycobacterium brisbanense*, and *Mycobacterium mageritense*, although some controversy about the classification of *M. mageritense* still exists [4, 5].

Difficult-to-treat infections caused by RGM are increasingly observed in clinical settings, especially by *M. abscessus* complex, which is considered one of the most resistant strains [3]. Additionally, inducible macrolide resistance, a recently recognized phenomenon, may restrict the therapeutic role of macrolides. This resistance may be related to the insufficient efficacy of clarithromycin-based treatment of *M. abscessus* subsp. *abscessus* infection, even when a particular isolate is initially shown to be sensitive to the drug [6, 7]. To date, only a small amount of data is available on antimicrobial susceptibilities of RGM isolated in Japan. Susceptibility of RGM to some antimicrobials can be tested in a limited number of reference laboratories, while an accurate identification of RGM at the species level is difficult for most of the clinical laboratories, resulting in limited susceptibility data as well.

The development of novel treatment options is necessary since the effective antimicrobial treatment options against RGM, in particular against *M. abscessus* subsp. *abscessus*, are limited [3]. Oral medications need to be investigated in addition to parenteral agents, since the long-term antimicrobial treatments are often required. Tigecycline, the first clinically available glycylcycline, has broad activity against multidrug-resistant bacteria, due to its low sensitivity to major mechanisms of tetracycline resistance [8]. Minimal inhibitory concentrations (MICs) of this drug were shown to be low against rapidly growing mycobacterial isolates tested throughout the world [9–14]. Tebipenem, a novel oral carbapenem that is approved for use in Japan, was recently showed to have potent in vitro activity against *Mycobacterium tuberculosis* [15], although the data are still not complete.

The purpose of this study was to examine the susceptibilities of RGM clinical isolates collected in Japan to 16 antimicrobial agents, including potential new agents, such as tigecycline and tebipenem. We placed particular emphasis on the correct identification of isolates at the species level, prior to the drug susceptibility testing.

## Methods

### Bacterial identification

Between December 2010 and August 2013, 71 strains of RGM were isolated from various clinical samples at the University of Tokyo Hospital, Japan. We identified these strains using matrix-assisted laser desorption/ionization time-of-flight mass spectrometry (MALDI-TOF MS) and the sequencing of three conserved genes (16S rRNA, *rpoB*, and *hsp65*). The MALDI Biotyper system and the Mycobacterium Library 1.0 database (Bruker Daltonics K.K., Kanagawa, Japan) were used for MALDI-TOF MS analyses.

### Antimicrobial susceptibility testing

Susceptibility testing was performed according to Clinical and Laboratory Standards Institute (CLSI) guidelines M24-A2 [16], using frozen broth microdilution panels. The final drug concentration ranges were as follows: 4 to 128 μg/mL of amikacin, 1 to 16 μg/mL of tobramycin, 0.12 to 16 μg/mL of tigecycline, 0.25 to 16 μg/mL of minocycline, 0.25 to 8 μg/mL of ciprofloxacin, 0.25 to 8 μg/mL of moxifloxacin, 0.25 to 32 μg/mL of clarithromycin, 1 to 16 μg/mL of azithromycin, 2 to 64 μg/mL of linezolid, 1 to 64 μg/mL of imipenem, 1 to 64 μg/mL of meropenem, 0.25 to 2 μg/mL of faropenem, 0.5 to 4 μg/mL of tebipenem, 4 to 128 μg/mL of cefmetazole, 2 to 64 μg/mL of cefepime, and 1/19 to 8/152 μg/mL of trimethoprim/sulfamethoxazole. Tigecycline was purchased from Selleckchem (Houston, TX, USA). Frozen broth microdilution panels containing antimicrobial agents in 100 μL of cation-supplemented Muellar-Hinton broth were custom-fabricated by Eiken Chemical Co., Ltd. (Tokyo, Japan). According to the CLSI guidelines [16], the inoculum was prepared so that the final concentrations were 1 × 10^5^ to 5 × 10^5^ CFU/mL. Strains were incubated at 37 °C. If microbial growth in the control sample was sufficient, MICs were measured at day 3. Otherwise, the incubation period was prolonged, and MICs were measured at day 4 or 5. For clarithromycin, MIC measurements were performed at day 3 and day 14 to detect inducible macrolide resistance. In this study, clarithromycin MICs were determined at day 14 of incubation. *M. fortuitum* ATCC 6841 was used as a quality control reference strain.

The MIC breakpoints, indicating susceptible, intermediate, and resistant strains, were interpreted according to the CLSI criteria for amikacin, tobramycin, minocycline, ciprofloxacin, moxifloxacin, clarithromycin, linezolid, imipenem, meropenem, and trimethoprim/sulfamethoxazole [16]. To date, there are no consensus breakpoints for tigecycline, azithromycin, faropenem, tebipenem, cefmetazole, and cefepime. Because of this, a modified susceptibility breakpoint of ≤4 μg/mL for tigecycline proposed by Petrini [17] was used in this study (Table 1).

**Table 1:** Antimicrobial agents and MIC breakpoints for rapidly growing mycobacteria

Antimicrobial agents	MIC^a^ (μg/mL) for category		
	Susceptible	Intermediate	Resistant
Amikacin	≤16	32	≥64
Tobramycin	≤2	4	≥8
Tigecycline	≤4		>4
Minocycline	≤1	2–4	≥8
Ciprofloxacin	≤1	2	≥4
Moxifloxacin	≤1	2	≥4
Clarithromycin	≤2	4	≥8
Linezolid	≤8	16	≥32
Imipenem	≤4	8–16	≥32
Meropenem	≤4	8–16	≥32
Sulfamethoxazole	≤38		≥76

*Abbreviation MIC* minimal inhibitory concentration

^a^Breakpoints of each drug, except for tigecycline, are based on the Clinical and Laboratory Standards Institute recommendations. Breakpoints for tigecycline were based on those proposed by Petrini [17]

## Results

### Bacterial isolates and species identification

Among the investigated strains, 46 were identified as *M. abscessus* complex, *M. chelonae*, and *M. fortuitum* group (including *M. fortuitum*, *M. mageritense*, *M. peregrinum*, *M. porcinum*, and *M. septicum*). Despite the sequencing of housekeeping genes (*rpoB* and *hsp65*) and the use of MALDI-TOF MS, we were unable to distinguish between *M. abscessus* subsp. *abscessus* and *M. abscessus* subsp. *bolletii* correctly. Therefore, we have described isolates belonging to *M. abscessus* species as *M. abscessus* complex. Of these three clinically important species/groups of RGM (46 isolates), four isolates were excluded: we were not able to cultivate three of them for susceptibility testing and the results of MALDI-TOF MS and gene sequencing were disparate for one isolate. Therefore, we included a total of 42 isolates in this study. They were collected from sputum/respiratory specimens (*n* = 35), wounds (*n* = 3), blood (*n* = 1), ascites (*n* = 1), lymph node aspirate (*n* = 1), and ocular discharge sample (*n* = 1).

Among the 42 isolates included in this study, 13 isolates were identified as *M. abscessus* complex, 12 as *M. chelonae*, nine as *M. fortuitum*, four as *M. mageritense*, two as *M. peregrinum*, one as *M. porcinum*, and one as *M. septicum*.

### Antimicrobial susceptibility

In this study, sufficient microbial growth in the growth control samples was observed after 72 h of incubation. The results of the susceptibility testing (MIC range, MIC_50_, MIC_90_, and percentage of susceptibility) to 16 antimicrobial agents are presented in Table 2. All isolates (*n* = 42) were highly susceptible to tigecycline, with MIC_50_ values of ≤0.12 μg/mL, MIC_90_ value of 0.25 μg/mL, and MIC range of ≤0.12–0.5 μg/mL.

**Table 2:** Results of susceptibility testing of 16 antimicrobial agents against RGM

RGM species (No. of isolates tested) and antimicrobial agent	MIC (μg/mL)			Percentage of isolates		
	Range	MIC_50_	MIC_90_	S	I	R
*M. abscessus* complex (13)						
Amikacin	8–16	16	16	100	0	0
Tobramycin	8– >16	16	>16	0	0	100
Tigecycline	≤0.12–0.5	0.25	0.5	100	-^a^	0
Minocycline	≤0.25– >16	>16	>16	15.4	15.4	69.2
Ciprofloxacin	4– >8	>8	>8	0	0	100
Moxifloxacin	2– >8	8	>8	0	7.7	92.3
Clarithromycin	≤0.25– >32	0.5	>32	61.5	7.7	30.8
Azithromycin	≤1– >16	16	>16	-	-	-
Linezolid	≤2–32	4	16	76.9	15.4	7.7
Imipenem	2–16	8	16	30.8	69.2	0
Meropenem	8–64	16	32	0	69.2	30.8
Faropenem	>2	>2	>2	-	-	-
Tebipenem	4– >4	>4	>4	-	-	-
Cefmetazole	8–64	32	32	-	-	-
Cefepime	32– >64	64	>64	-	-	-
Sulfamethoxazole	>152	>152	>152	0	-	100
*M. chelonae* (12)						
Amikacin	≤4–64	16	16	91.7	0	8.3
Tobramycin	≤1–4	2	4	83.3	16.7	0
Tigecycline	≤0.12–0.25	≤0.12	0.25	100	-	0
Minocycline	8– > 16	16	>16	0	0	100
Ciprofloxacin	≤0.25– >8	8	>8	16.7	8.3	75.0
Moxifloxacin	≤0.25–8	2	4	16.7	41.7	41.7
Clarithromycin	≤0.25	≤0.25	≤0.25	100	0	0
Azithromycin	≤1–2	≤1	2	-	-	-
Linezolid	≤2–8	≤2	8	100	0	0
Imipenem	≤1–16	4	8	58.3	41.7	0
Meropenem	≤1–64	64	64	8.3	8.3	83.3
Faropenem	≤0.25– >2	>2	>2	-	-	-
Tebipenem	≤0.5– >4	>4	>4	-	-	-
Cefmetazole	≤4– >128	>128	>128	-	-	-
Cefepime	≤2–64	16	32	-	-	-
Sulfamethoxazole	38– >152	>152	>152	8.3	-	91.7
*M. fortuitum* (9)						
Amikacin	≤4	≤4	≤4	100	0	0
Tobramycin	16– >16	>16	>16	0	0	100
Tigecycline	≤0.12	≤0.12	≤0.12	100	-	0
Minocycline	≤0.25– >16	≤0.25	>16	55.6	0	44.4
Ciprofloxacin	≤0.25	≤0.25	≤0.25	100	0	0
Moxifloxacin	≤0.25	≤0.25	≤0.25	100	0	0
Clarithromycin	4– >32	32	>32	0	11.1	88.9
Azithromycin	>16	>16	>16	-	-	-
Linezolid	4–16	8	16	88.9	11.1	0
Imipenem	≤1–8	2	8	66.7	33.3	0
Meropenem	4–8	4	8	66.7	33.3	0
Faropenem	>2	>2	>2	-	-	-
Tebipenem	1–4	2	4	-	-	-
Cefmetazole	8–16	16	16	-	-	-
Cefepime	>64	>64	>64	-	-	-
Sulfamethoxazole	76– >152	>152	>152	0	-	100
*M. mageritense* (4)						
Amikacin	≤4– >128	128	>128	25.0	0	75.0
Tobramycin	>16	>16	>16	0	0	100
Tigecycline	≤0.12	≤0.12	≤0.12	100	-	0
Minocycline	2– >16	2	>16	0	50.0	50.0
Ciprofloxacin	≤0.25–1	≤0.25	1	100	0	0
Moxifloxacin	≤0.25	≤0.25	≤0.25	100	0	0
Clarithromycin	32– >32	>32	>32	0	0	100
Azithromycin	16– >16	16	>16	-	-	-
Linezolid	≤2–16	≤2	16	75.0	25.0	0
Imipenem	≤1–2	≤1	2	100	0	0
Meropenem	≤1–4	≤1	4	100	0	0
Faropenem	2– >2	2	>2	-	-	-
Tebipenem	1–4	2	4	-	-	-
Cefmetazole	≤4–8	≤4	8	-	-	-
Cefepime	>64	>64	>64	-	-	-
Sulfamethoxazole	≤19– >152	152	>152	25.0	-	75.0
*M. peregrinum* (2)						
Amikacin	≤4	≤4	≤4	100	0	0
Tobramycin	4–8	4	8	0	50.0	50.0
Tigecycline	≤0.12	≤0.12	≤0.12	100	-	0
Minocycline	>16	>16	>16	0	0	100
Ciprofloxacin	≤0.25	≤0.25	≤0.25	100	0	0
Moxifloxacin	≤0.25	≤0.25	≤0.25	100	0	0
Clarithromycin	≤0.25	≤0.25	≤0.25	100	0	0
Azithromycin	≤1– >16	≤1	>16	-	-	-
Linezolid	4–8	4	8	100	0	0
Imipenem	≤1–2	≤1	2	100	0	0
Meropenem	2–4	2	4	100	0	0
Faropenem	>2	>2	>2	-	-	-
Tebipenem	≤0.5–1	≤0.5	1	-	-	-
Cefmetazole	≤4–8	≤4	8	-	-	-
Cefepime	>64	>64	>64	-	-	-
Sulfamethoxazole	38– >152	38	>152	50.0	-	50.0

*Abbreviations RGM* rapidly growing mycobacteria, *MIC* minimal inhibitory concentration, *S* susceptible, *I* intermediate, *R* resistant

^a^not applicable

### Antimicrobial susceptibility patterns of RGM

#### *M. abscessus* complex

All isolates belonging to *M. abscessus* complex (*n* = 13) were susceptible to amikacin. However, they were all resistant to tobramycin and trimethoprim-sulfamethoxazole and not susceptible to fluoroquinolones (both moxifloxacin and ciprofloxacin). Seventy percent of the isolates and none of them were susceptible to imipenem and meropenem, respectively. Around two-thirds of the isolates were susceptible to clarithromycin and linezolid (Table 2).

#### *M. chelonae*

Among *M. chelonae* strains (*n* = 12), all isolates were susceptible to clarithromycin and linezolid, azithromycin MICs were shown to be ≤2 μg/mL for all isolates. The majority of *M. chelonae* isolates were susceptible to aminoglycosides: 83% were susceptible to tobramycin, used predominantly in the treatment of *M. chelonae* infections [16]. All *M. chelonae* isolates were resistant to minocycline, while they were susceptible to another tetracycline derivative, tigecycline. Susceptibility to different carbapenems was diverse, with 58% of isolates susceptible to imipenem and only 8% susceptible to meropenem (Table 2).

#### *M. fortuitum*

Similar to *M. abscessus* complex, among *M. fortuitum* strains (*n* = 9), all isolates were susceptible to amikacin and resistant to tobramycin. Many antimicrobial agents showed activity or had low MICs in vitro against *M. fortuitum*. All *M. fortuitum* isolates were susceptible to fluoroquinolones (ciprofloxacin and moxifloxacin), and around two-thirds of isolates were susceptible to imipenem and meropenem. Furthermore, the MIC_90_ values of tebipenem and cefmetazole against *M. fortuitum* isolates were lower compared with the values against *M. abscessus* complex and *M. chelonae*. All isolates were found to be susceptible to tigecycline, although 44% were resistant to minocycline. However, all *M. fortuitum* isolates were not susceptible to clarithromycin and had high MICs (>16 μg/mL) to azithromycin (Table 2).

### Other *M. fortuitum* group

Other *M. fortuitum* group isolates, *M. mageritense* (*n* = 4)*, M. peregrinum* (*n* = 2)*, M. porcinum* (*n* = 1)*,* and *M. septicum* (*n* = 1), generally had good sensitivity to fluoroquinolones, carbapenems (including tebipenem), cefmetazole, and linezolid; however, only a small number of these strains had been included in this study. Additionally, similar to *M. fortuitum,* all these isolates, except *M. peregrinum,* showed poor sensitivity to clarithromycin. *M. fortuitum* group isolates were not susceptible to minocycline, while they all were susceptible to tigecycline (Table 2).

## Discussion

In this study, conducted in Japan, antimicrobial susceptibility patterns of different species of RGM were found to vary. However, rapidly growing mycobacterial strains, regardless of species, showed good sensitivity to tigecycline. Although a susceptibility breakpoint of ≤4 μg/mL was used for tigecycline [17] in this study, the growth of all isolates was inhibited by tigecycline at very low MICs, that is, ≤0.5 μg/mL. To date, most of the RGM worldwide remain sensitive to tigecycline in vitro. The MIC values of tigecycline against a total of 122 rapidly growing mycobacterial isolates (including 50 tetracycline-resistant isolates) collected in USA [9], 40 isolates obtained in Taiwan [10], 25 isolates collected in Turkey [11], 160 isolates in Taiwan [12], and 57 isolates obtained in Korea [13] were ≤4 μg/mL. The MIC ranges in the first four studies were reported to be ≤0.06–1 μg/mL, 0.064–2 μg/mL, 0.12–1 μg/mL, and 0.0625–4 μg/mL, respectively; the MIC range was not reported in the Korean study. However, in a recent study conducted in China, it was shown that tigecycline MIC values against two or three of 73 RGM isolates were somewhat high, with an MIC of 8 μg/mL [14].

It was previously reported that tigecycline may have a promising role in the treatment of multi-drug resistant RGM infections, not only in vitro but also in vivo. A study following 52 patients with *M. abscessus* and *M. chelonae* infections, where prior therapy attempts had failed, showed that tigecycline-containing regimens administered for ≥1 month resulted in clinical improvement in more than 60% of the patients [18]. However, both CLSI and European Committee on Antimicrobial Susceptibility Testing (EUCAST) have not defined tigecycline MIC breakpoints for RGM yet. Additionally, tigecycline has not been approved for the treatment of mycobacterial infections, demonstrating a need for further clinical studies and appropriate approvals, in order to develop new treatment options.

Macrolides are one of the most important drugs used for the treatment of RGM. However, we observed that a relatively large number of isolates showed resistance to macrolides, with important differences in susceptibility patterns among species; the obtained rates of clarithromycin-susceptible strains were 0, 62, and 100% of *M. fortuitum*, *M. abscessus* complex, and *M. chelonae*, respectively. Additionally, *M. mageritense* strain, which comprises 10% of all isolates in this study, shows intrinsic resistance to macrolides [2]. Inducible resistance to macrolides can occur during the application of macrolide-containing regimen during the treatment of infections caused by several RGM species. The erythromycin ribosome methyltransferase genes, *erm*(38) and *erm*(41), have been reported to confer inducible macrolide resistance to *M. fortuitum* and *M. abscessus* subsp. *abscessus*, respectively [6, 19], while *erm*(41) is absent from *M. chelonae* [6]. Inducible resistance may be at least partially responsible for the difficulties observed during the treatment of *M. abscessus* infections [20].

Previously, approximately 80% of *M. fortuitum* samples were shown to be susceptible to clarithromycin [19], which was not observed in this study. In a study conducted in Spain, of 89 *M. fortuitum* clinical isolates, 75 (84.3%) were shown to harbor the *erm* gene, while 42 (47.2%) were not susceptible to clarithromycin [21]. Regional differences in clarithromycin susceptibility and the prevalence of *erm* gene in the clinical isolates of *M. fortuitum* may exist. Further studies investigating this issue are necessary.

Azithromycin susceptibility breakpoint is not defined by the CLSI and EUCAST, and CLSI lists clarithromycin as a class representative of a new generation of macrolides, which includes azithromycin, for the RGM susceptibility testing [16]. However, to date, no conclusive results about the equivalence of the clinical effects of clarithromycin and azithromycin therapies in the patients with *M. abscessus* infections were obtained. In the experimental models of *M. abscessus* subsp. *abscessus* and *M. abscessus* subsp. *massiliense* infections, clarithromycin induced *erm*(41) expression, leading to a higher induction of macrolide resistance compared with the effects of azithromycin on *M. abscessus* subsp. *abscessus*. As a result, clarithromycin was shown to have a lower effect against *M. abscessus* subsp. *abscessus* in vitro and in vivo compared with azithromycin, while both of these macrolides showed comparable effects against *M. abscessus* subsp. *massiliense* infections [7]. In order to ensure the efficient treatment using macrolides, a correct identification of subspecies of *M. abscessus* complex (including *M. abscessus* subsp. *abscessus* and *M. abscessus* subsp. *massiliense*) is clinically important, although we have not performed this identification, as it is technically cumbersome.

Carbapenem is one of the available treatment options for RGM infections. Imipenem was demonstrated to be more efficient against *M. abscessus* complex, *M. chelonae*, and *M. fortuitum* than meropenem in this study. In Japan, faropenem (an oral penem) and tebipenem (an oral carbapenem) are approved for clinical use. Tebipenem MIC values were shown to be ≤4 μg/mL against all isolates belonging to *M. fortuitum* group (including *M. fortuitum*, *M. mageritense, M. peregrinum*, *M. porcinum*, and *M. septicum*) in this study, while the MICs of this compound against the majority of *M. abscessus* complex and *M. chelonae* isolates were >4 μg/mL (Table 2). Since concentrations higher than 4 μg/mL are achievable in human serum at therapeutic doses [15], tebipenem may represent an orally available drug of choice for the treatment of infections caused by *M. fortuitum* group strains. Further clinical studies are necessary to evaluate these new agents against difficult-to-treat RGM infections.

Many RGM strains, with the exception of *M. mageritense,* showed sensitivity to amikacin. Tobramycin was active only against *M. chelonae* and *M. septicum*. CLSI discourages clinical use of tobramycin for the treatment of infections caused by *M. abscessus* or *M. fortuitum* group. As the information on *M. mageritense* susceptibility to amikacin remains limited, it should be further investigated.

In this study, we used *M. fortuitum* ATCC 6841 as a control strain, which represents one of the potential limitations of this study, as the current CLSI-recommended quality control strain is *M. peregrinum* ATCC 700686.

## Conclusions

We performed susceptibility testing of 16 antimicrobial agents against 42 isolates of *M. abscessus* complex, *M. chelonae*, and *M. fortuitum* group collected in Japan, which were correctly identified using MALDI-TOF MS and 16S rRNA, *rpoB*, and *hsp65* gene sequencing. Tigecycline was able to inhibit the growth of all tested isolates at very low MICs of ≤0.5 μg/mL, regardless of species and tetracycline resistance. Tebipenem showed MIC values of ≤4 μg/mL against all isolates of *M. fortuitum* group. We have showed that the patterns of antimicrobial susceptibility clearly differ among RGM species, and that the correct identification of RGM species (and even subspecies) and antimicrobial susceptibility testing, including the identification of novel agents, are important for the treatment of RGM infections.

## Abbreviations

RGM: Rapidly growing mycobacteria
MICs: Minimal inhibitory concentrations
MALDI-TOF MS: Matrix-assisted laser desorption/ionization time-of-flight mass spectrometry
CLSI: Clinical and Laboratory Standards Institute
EUCAST: European Committee on Antimicrobial Susceptibility Testing
